# Imbalance of Circulating Tfh/Tfr Cells in Patients With Parkinson's Disease

**DOI:** 10.3389/fneur.2020.572205

**Published:** 2020-10-02

**Authors:** Xiuzhen Zhao, Tao Jin, Chao Zheng, Di Ma, Ying Zhang

**Affiliations:** ^1^Department of Neurology and Neuroscience Center, The First Hospital of Jilin University, Changchun, China; ^2^Department of Neurology, Jining No. 1 People's Hospital, Shandong, China

**Keywords:** Parkinson's disease, follicular helper T cell, follicular regulatory T cell, pathogensis, immune inflammation

## Abstract

**Background:** Follicular helper T (Tfh) cells and follicular regulatory T (Tfr) cells are essential for B cell differentiation, germinal center formation, and humoral immune responses. Immunity and inflammation have been thought to be involved in Parkinson's disease (PD). In this study, we aimed to identify whether circulating Tfh and Tfr (cTfh and cTfr) cells contribute to PD.

**Methods:** Thirty-nine PD patients and 26 health controls (HCs) were enrolled. The numbers of cTfh (CD4^+^CXCR5^+^PD-1^+^) cells and cTfr (CD4^+^CXCR5^+^CD25^hi^CD127^low^) cells were analyzed via flow cytometry. The serum concentrations of interleukin (IL)-4, IL-10, IL-21, and transforming growth factor (TGF)-β were examined by cytometric bead array.

**Results:** The percentage of cTfh cells among CD4^+^ T cells in PD patients was significantly higher than that in HCs [3.68% (2.64–5.70%) vs. 1.94% (1.32%−2.99%), *P* < 0.001], while the percentage of cTfr cells among CD4^+^ T cells in PD patients was slight decreased but without significance [1.05% (0.62–1.54%) vs. 1.3% (0.63–1.90%), *P* > 0.05]. The percentage of CD19^+^ B cells in peripheral blood mononuclear cells was significantly lower in PD patients than in HCs [5.35% (4.13–9.38%) vs. 8.68% (5.61–12.93%), *P* = 0.014]. The serum concentrations of IL-4, IL-10, IL-21, and TGF-β in PD patients did not differ significantly from those in HCs (*P* > 0.05). There was a positive trend of the correlation between the number of cTfh and the serum IL-4 concentrations in PD patients (*P* = 0.032, *r* = 0.353). There was a positive trend of the correlation between the number of cTfr and the serum IL-10 concentrations in PD patients (*P* = 0.047, *r* = 0.328), A positive trend of the correlation were found for the serum concentration of IL-21 with H-Y stage (*r* = 0.356, *P* = 0.026) and UPDRS-III score (*r* = 0.347, *P* = 0.030).

**Conclusions:** These results indicate that an imbalance of cTfh and cTfr cells may be involved in the chronic progression of PD, and IL-21 may be a biomarker for monitoring the severity of this disease.

## Introduction

Parkinson's disease (PD), also known as tremor paralysis, is a common neurological degenerative disease in the elderly. The basic pathological changes of PD are the degeneration and loss of dopaminergic neurons in brain substantia nigra. Eosinophilic inclusion bodies appear in the remaining neurons and are called Lewy bodies. Alpha-synuclein (α-syn) is known to be the main structural component of the Lewy bodies (1). In 2003, Braak et al. proposed the Braak staging system for sporadic PD, which divided the pathological changes of PD into six stages according to the pathological diffusion sequence of α-syn (2). This theory provided an anatomical basis for the motor and non-motor symptoms of PD. Some researchers believed that α-syn accumulates in neurons and propagates in a prion-like manner, which is involved in the aggravation of PD (3). However, the detailed mechanism remains unclear.

In 1988, McGeer et al. found that activated microglia were present around the degenerative and necrotic dopaminergic neurons in the brain of PD patients on autopsy (4). Since then, the immune system and inflammation reaction have been considered to play roles in the pathogenesis of PD. Chen et al. injected the IgG extracted from the serum of PD patients into the right substantia nigra area of adult rats and observed obvious dopaminergic neuron loss and microglia infiltration in the injected side compared to the uninjected side (5). This suggests that abnormal pathogenic antibodies may be present in PD patients. Combined with the Braak staging theory, we speculate that abnormally aggregated α-syn may act as an autoantigen to continuously activate the body's autoimmune response, resulting in an immune stress state in the body, which may be one reason for the chronic and persistent progression of PD.

Follicular helper T (Tfh) cells, a special CD4^+^ T-cell subset, were discovered by Schaerli et al. in 2000. They are derived from the tonsil tissue and localized in the lymphoid follicles. Tfh cells are essential in the differentiation of B cells into high-affinity plasma cells, the formation of germinal centers, and immunoglobulin class switching. They can also maintain the body's long-term humoral immune response (6–8). In contrast, follicular regulatory T (Tfr) cells, which were recently defined, can suppress the reactions of Tfh cells and B cells in the germinal center and inhibit the sustained immune activation state (9, 10). The balance between Tfh and Tfr cells plays an important role in maintaining the body's immune homeostasis. Tfh cells and Tfr cells in the peripheral circulation express the same surface molecules as the respective cell types in lymphoid tissues and have the same functional characteristics. Imbalance of circulating Tfh and Tfr (cTfh and cTfr) cells has been shown in autoimmune diseases such as rheumatoid arthritis (11), myasthenia gravis (12), ulcerative colitis (13), and renal allograft dysfunction (14). To date, few studies have investigated the balance of cTfh and cTfr cells in PD.

In the present study, we measured the levels of Tfh cells and Tfr cells as well as the related cytokines in PD patients. Our results suggest that changes in relative cTfh and cTfr cell numbers may cause an immune responsive state in PD patients, contributing to the pathogenesis of PD.

## Materials and Methods

### Patients

The study was approved by the ethics committee of the First Hospital of Jilin University. Fasting blood samples were obtained from PD patients (*n* = 39) and age- and gender-matched healthy controls (HCs, *n* = 26). All subjects provided informed consent according to the Declaration of Helsinki. The enrolled PD patients were diagnosed and treated in the Department of Neurology of the First Hospital of Jilin University during 2017-2018. The disease was evaluated according to the clinical diagnostic criteria for PD put forth by the Movement Disorders Association (MDS) in 2015 (15). The exclusion criteria included recent infection symptoms or suspected infection; usage of any anti-inflammatory drugs (such as NSAIDs), hormones, and immunosuppressants in the past 3 months; autoimmune diseases (such as systemic lupus erythematosus, rheumatoid arthritis, Sjogren's syndrome, myasthenia gravis, multiple sclerosis, etc.); severe digestive, circulatory, endocrine, and hematological disorders; and familial PD. The third part of the Unified Parkinson's Disease Rating Scale (UPDRS-III) (16) and modified Hoehn-Yahr stage scale (H-Y stage) (17) were used to evaluate the motor symptoms and clinical staging of patients. The Non-Motor Symptoms Scale (NMSS) was used to assess the severity of patients' nonmotor symptoms (18). According to H-Y stage, PD patients were divided into two subgroups: stage I-II is early PD, and more than stage II is defined as middle-advanced PD. The demographic and clinical characteristics of the study participants are summarized in Table 1.

**Table 1:** Demographic and clinical characteristics of enrolled PD patients and HCs.

	**HCs**	**PD patients**	**Early PD patients**	**Middle-advanced PD patients**
Number	26	39	28	11
Age (years)	60.77 ± 6.77	61.49 ± 5.79	61.71 ± 5.98	60.91 ± 5.47
Gender (male/female)	13/13	20/19	16/12	4/7
BMI (kg/m^2^)	24.09 ± 2.34	23.92 ± 2.96	23.84 ± 2.59	24.10 ± 3.88
Disease duration (years)	–	3 (1–8)	3 (1–6)	8 (3–10)^*^
Hoehn-Yahr stage	–	1.5 (1–3)	1.25 (1–1.5)	3 (3–3)^*^
UPDRS-III score	–	19 (14–28)	16 (12–20)	35 (26–47)^*^
NMSS score	–	48.18 ± 25.44	42.79 ± 20.43	61.91 ± 32.29^*^
Levodopa dosage (mg)	–	300(150–400)	300(150–300)	400(300–550)^*^

*Data are shown as mean ± standard deviation or median (interquartile range). BMI, Body Mass Index*.

* *P < 0.05 between the early PD and middle-advanced PD groups*.

### Cell Isolation and Flow Cytometry

**Figure 1:**
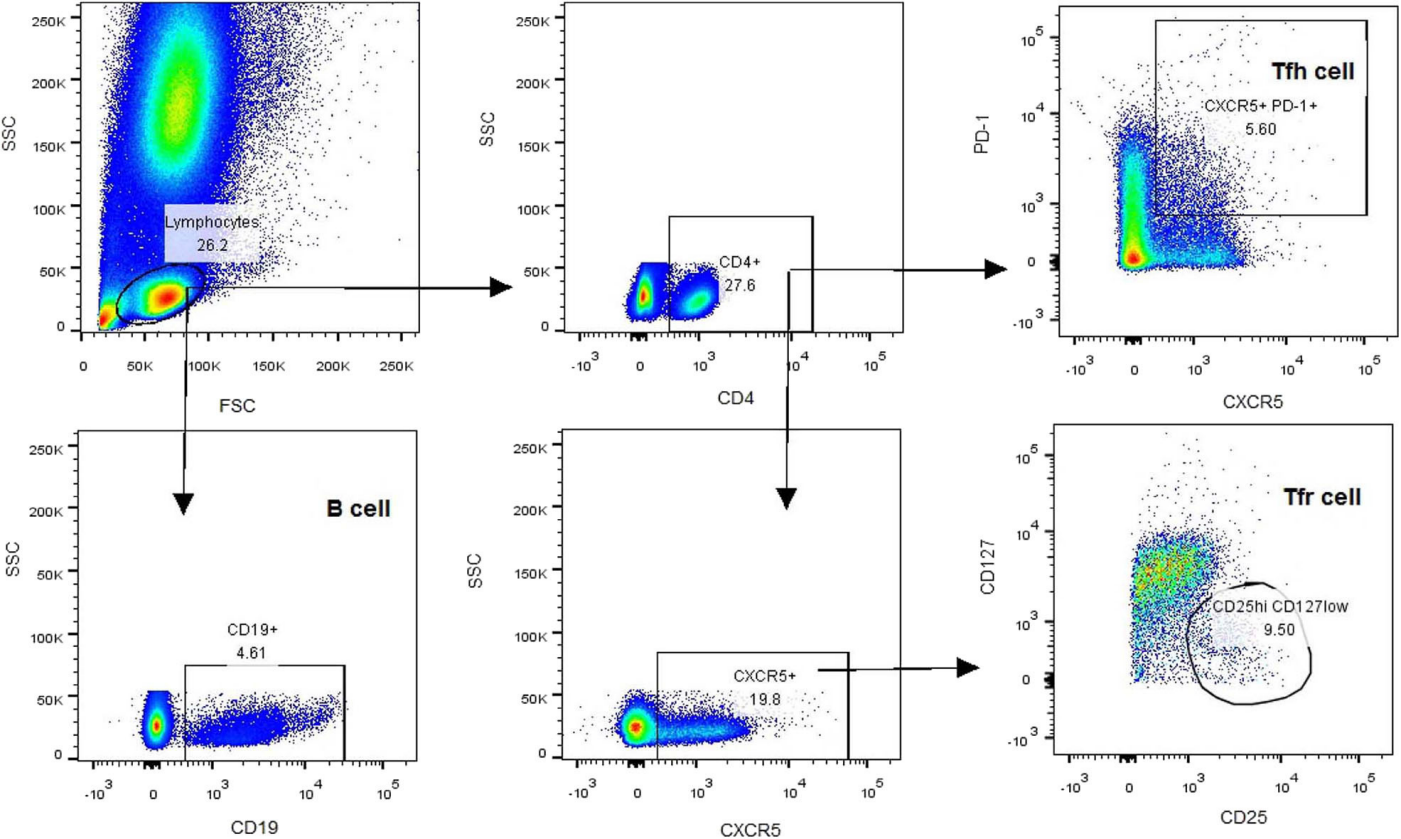
Flow cytometry sorting of cTfh, cTfr, and CD19^+^B cells.

All participants were taken blood samples at 7–9 am, empty stomach, and adequate sleep time, in the on period of medication. Heparin-anticoagulated whole blood samples were collected to determine the T-cell subsets. Peripheral blood mononuclear cells (PBMCs) were separated within 4 h by density gradient centrifugation with red blood cell lysis buffer (Solarbio, China). Aliquots of 1 × 10^6^ PBMCs were re-suspended in PBS and stained with antibodies at room temperature for 30 min. The antibodies included V500-conjugated anti-CD4 antibody, Alexa Fluor 647-conjugated anti-CXCR5 antibody, PerCP-Cy5.5-conjugated anti-PD-1 antibody, FITC-conjugated anti-CD25 antibody, V451-conjugated anti-CD127 antibody, and PE-conjugated anti-CD19 antibody. After staining, PBMCs were washed three times and analyzed using a BD FACSAria ^TM^ II Cell Sorter (BD, USA). The gating strategy was first based on the forward/side scatter, followed by CD4 and CXCR5 positivity. After collection of CD4^+^CXCR5^+^ T cells, the frequencies of cTfh (CD4^+^CXCR5^+^PD-1^+^) and cTfr (CD4^+^CXCR5^+^CD25^hi^CD127^low^) cells were calculated based on PD-1 and CD25/CD127 expression, respectively. The percentage of B cells (CD19^+^) was also calculated (Figure 1).

### Measurement of Serum IL-4, IL-10, IL-21, and TGF-β Concentrations

Serum samples were collected and stored at −80°C until analysis. The serum concentrations of IL-4, IL-10, IL-21, and TGF-β were determined by cytometric bead array (CBA) with human CBA Flex Sets, according to the manufacturer's protocols (BD, USA).

### Statistical Analysis

All data were analyzed using SPSS 22.0 statistical software (SPSS, Inc., USA). The measurement data were tested for normality. For data with a normal distribution, Values are expressed as mean ± standard deviation, and two independent sample *t*-tests were performed for comparisons. For data with a non-normal distribution, values are expressed as the median (interquartile range), and the Mann-Whitney U test was performed for comparisons. Spearman correlation analysis was performed to identify correlations between variables. *P* < 0.05 was considered statistically significant.

## Results

### Characteristics of Enrolled Subjects

A total of 39 patients with PD and 26 age- and gender-matched HCs were recruited. The demographic and clinical characteristics of the subjects are described in Table 1. There were no significant differences in age, gender, or body mass index (BMI) between the PD and HC groups. Compared to the patients in the early PD group, patients in the middle-advanced PD group had a significantly increased disease duration, H-Y stage, UPDRS-III score, and NMSS score (*P* < 0.05). The difference of UPDRS-III score between early PD group and middle-advanced PD group were statistically significant [16 (12–20), 95%CI (14, 18) vs. 35 (26–47), 95%CI (26, 47), *P* < 0.0001, Supplementary Figure 1].

### Altered Frequencies of cTfh, cTfr, and B Cells in PD Patients

**Figure 2:**
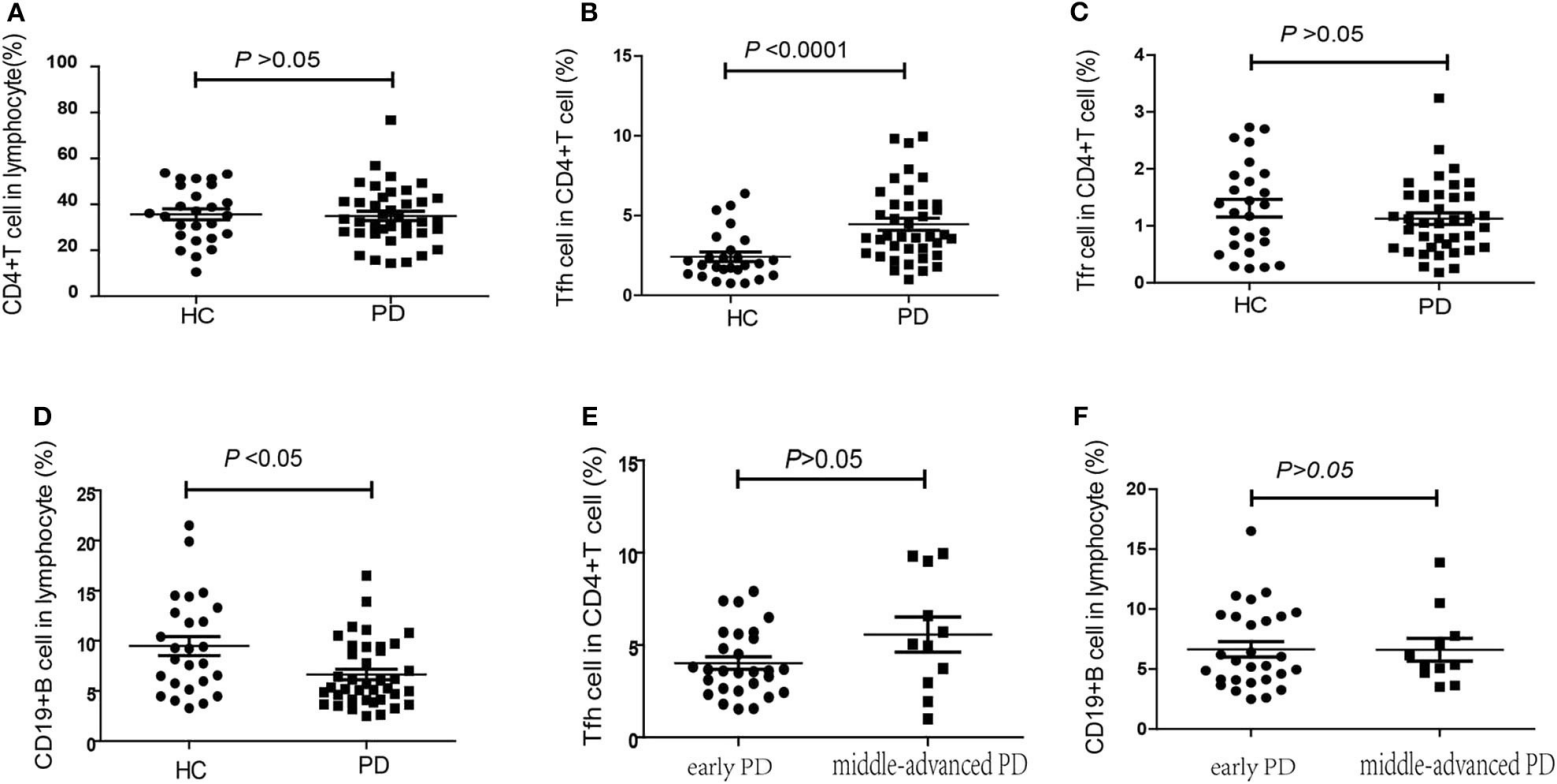
The percentage of cTfh, cTfr and B cells in PD patients and HCs. **(A)** The comparison of the CD4^+^ T cells between PD patients and HCs (*P* > 0.05). **(B)** The comparison of the cTfh cells between PD patients and HCs. The cTfh cells among CD4^+^ T cells was significantly higher in PD patients (*P* < 0.0001). **(C)** A comparison of the cTfr cells in PD patients and HCs, but it was without significant difference (*P* > 0.05). **(D)** A comparison of the CD19^+^ B cells in PD patients and HCs. The CD19^+^ B cells significantly lower in PD patients (*P* < 0.05). **(E)** The comparison of the cTfh cells between early PD group and middle-advanced PD group (*P* > 0.05). **(F)** A comparison of the cTfr cells in early PD group and middle-advanced PD group (*P* > 0.05).

According to the expression patterns of PD-1 and CD25/CD127, CD4^+^CXCR5^+^ cells in the peripheral blood were classified as cTfh (CD4^+^CXCR5^+^PD-1^+^) cells and cTfr (CD4^+^CXCR5^+^CD25^hi^CD127^low^) cells. Compared to that in HCs, the percentage of CD4^+^ T cells among lymphocytes was slightly lower in PD patients, but this difference was without statistical significance [32.60% (27.60–41.95%), 95%CI (29.85, 40.10%) vs. 35.65% (26.50–48.4%), 95%CI (29.05, 41.14%), *P* = 0.703, Figure 2A]. The percentage of cTfh cells among CD4^+^ T cells was significantly higher in PD patients than in HCs [3.68% (2.64–5.70%), 95%CI (3.30, 5.04%) vs. 1.94% (1.32–2.99%), 95%CI (1.61, 2.37%), *P* < 0.0001, Figure 2B]. However, there was no significant difference between the early PD group and middle-advanced PD group [3.60% (2.54–5.54%), 95%CI (3.11, 4.50%) vs. 5.04% (2.96–9.54%), 95%CI (2.96, 9.54%), *P* = 0.138, Figure 2E]. The percentage of cTfr cells among CD4^+^ T cells appeared to be lower in PD patients than in HCs, but the difference was without statistically significant[1.05% (0.62–1.54%) 95%CI (0.79, 1.30%) vs. 1.3% (0.63–1.90%), 95%CI (0.72, 1.70%), *P* = 0.366, Figure 2C].

In our study, we also found that the percentage of CD19^+^ B cells among PBMCs was significantly lower in PD patients than in HCs [5.35% (4.13–9.38%), 95%CI (4.10, 7.01%) vs. 8.68% (5.61–12.93%), 95%CI(6.49, 11.80%), *P* = 0.014, Figure 2D]. However, similar to cTfh cells, no significant difference was found between the early PD group and middle-advanced PD group [5.48% (4.10–9.40%), 95%CI (4.37, 8.67%) vs. 5.35% (4.7–7.76%), 95%CI (4.7, 7.76%), *P* = 0.888, Figure 2F].

### Correlations of cTfh Percentage, cTfr Percentage, and cTfh/cTfr Ratio With Clinical Symptoms

**Figure 3:**
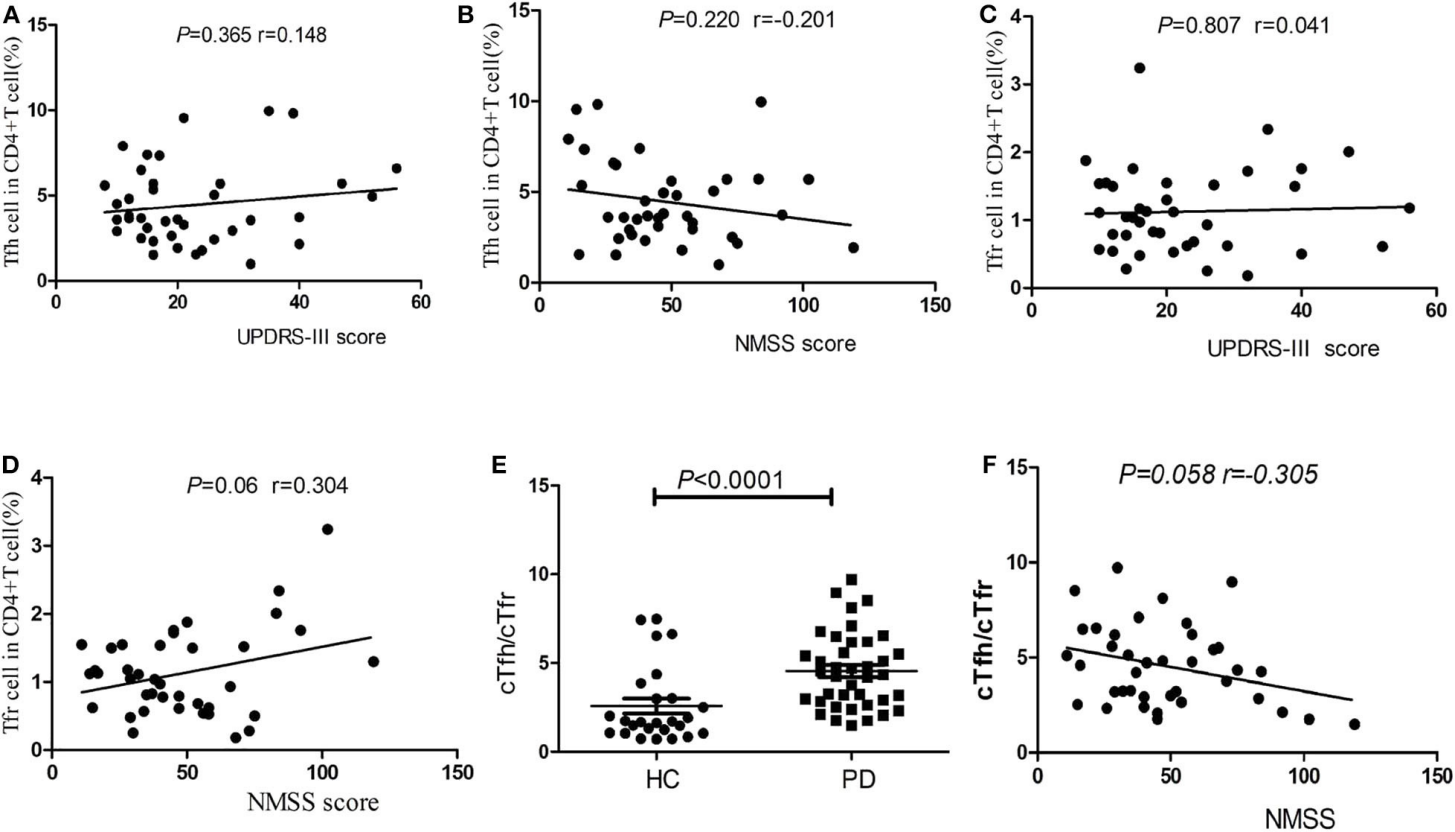
Correlation analysis of cTfh, cTfr and cTfh/cTfr for clinical symptoms. **(A)** The correlation analysis of the percentages of cTfh cells in PD patients with disease severity (UPDRS-III score) (*P* > 0.05). **(B)** The correlation analysis of the percentages of cTfh cells in PD patients with nonmotor symptoms (NMSS) (*P* > 0.05). **(C)** The correlation analysis of the percentages of cTfr cells in PD patients with disease severity (UPDRS-III score) (*P* > 0.05). **(D)** The correlation analysis of the percentages of cTfr cells in PD patients with nonmotor symptoms (NMSS) (*P* > 0.05). **(E)** A comparison of the cTfh/cTfr cells in PD patients and HCs. The cTfh/cTfr ratio in PD patients was significantly higher than that in HCs (*P* < 0.0001). **(F)** The correlation of the cTfh/cTfr ratio and NMSS in PD patients showed no statistical significance (*P* = 0.058).

No significant correlations were found between the percentages of cTfh cells or cTfr cells in PD patients with patients' age, disease duration, disease severity (UPDRS-III score), or nonmotor symptoms (NMSS) (*P* > 0.05, Figures 3A–D). However, the cTfh/cTfr ratio in PD patients was significantly higher than that in HCs [4.34 (2.84–6.18), 95%CI (3.21, 5.12) vs. 1.68 (1.05–3.21), 95%CI (1.30, 2.50), *P* < 0.0001, Figure 3E]. The correlation of the cTfh/cTfr ratio and NMSS in PD patients showed no statistical significance (*P* = 0.058, Figure 3F).

### Serum Concentrations of IL-4, IL-10, IL-21, and TGF-β in PD Patients

**Figure 4:**
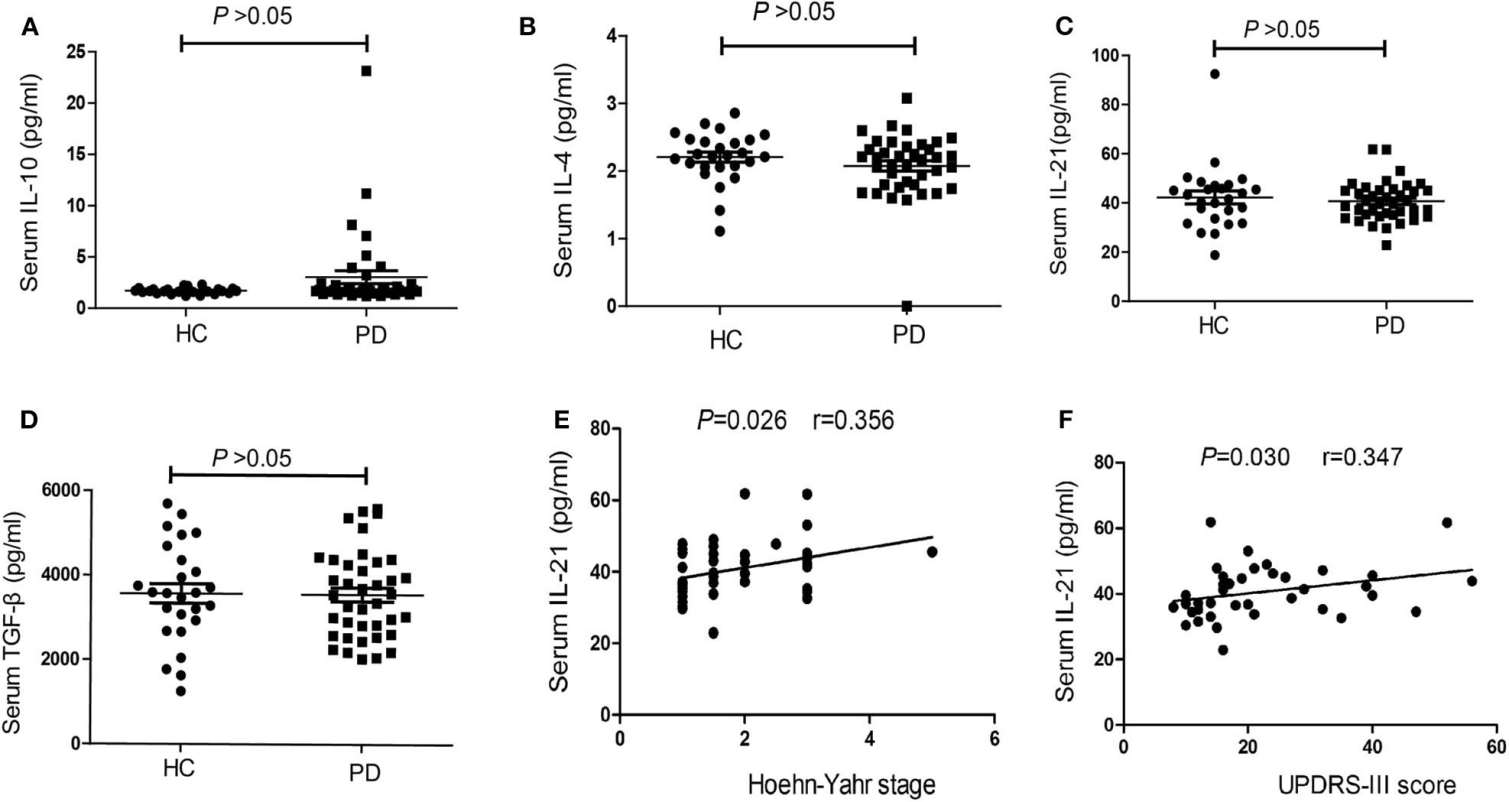
The concentrations and correlation analysis of IL-4, IL-10, IL-21, and TGF-β in PD patients. **(A)** The comparison of the IL-10 between PD patients and HCs (*P* > 0.05). **(B)** A comparison of the IL-4 in PD patients and HCs (*P* > 0.05). **(C)** The comparison of the IL-21between PD patients and HCs (*P* > 0.05). **(D)** A comparison of the TGF-β in PD patients and HCs (*P* > 0.05). **(E)** The correlation analysis of the serum IL-21 concentrations in PD patients with H-Y stage (*P* < 0.05). **(F)** The correlation analysis of the serum IL-21 concentrations in PD patients with UPDRS-III score (*P* < 0.05).

CBA measurements showed that the serum concentrations ofIL-10did not differ significantly between PD patients and HCs [1.76 (1.52–2.37), 95%CI (1.63, 1.98) vs. 1.68 (1.57–1.86), 95%CI (1.61, 1.81), *P* = 0.278, Figure 4A], the serum concentrations of IL-4 also did not differ significantly between PD patients and HCs [2.17 (1.80–2.36), 95%CI (1.97, 2.25) vs. 2.22 (2.06–2.46), 95%CI (2.11, 2.43), *P* = 0.172, Figure 4B]. We have not found the significant difference in the serum concentrations of IL-21 between PD patients and HCs [39.55 (35.15–45.21), 95%CI (36.75, 43.88) vs. 42.42 (33.15–47.15), 95%CI (37.38, 45.68), *P* = 0.588, Figure 4C], we have also not found the significant difference in the serum concentrations of TGF-β between PD patients and HCs [3,545.86 (2,612.3–4,333.03), 95%CI (2,971.69, 3,899.70) vs. 3,580.06 (2,868.06–4,441.79), 95%CI (3,202.30, 3948.81), *P* = 0.841, Figure 4D].

### Correlations of Serum IL-4, IL-10, IL-21, and TGF-β Concentrations With Clinical Symptoms

We also performed correlation analyses to identify significant associations between serum IL-4, IL-10, IL-21, and TGF-β concentrations and clinical symptoms. The results showed no significant correlations between serum IL-4, IL-10, and TGF-β concentrations in PD patients and patients' age, disease duration, disease severity (UPDRS-III score), and nonmotor symptoms (NMSS) (*P* > 0.05). However, there was a positive trend of the correlation between IL-21 level and H-Y stage (*r* = 0.356, *P* = 0.026, Figure 4E), as well as UPDRS-III score (*r* = 0.347, *P* = 0.030, Figure 4F).

### Correlations of cTfh Percentage and cTfr Percentage With Serum IL-4, IL-10, IL-21, and TGF-β Concentrations in PD Patients

**Figure 5:**
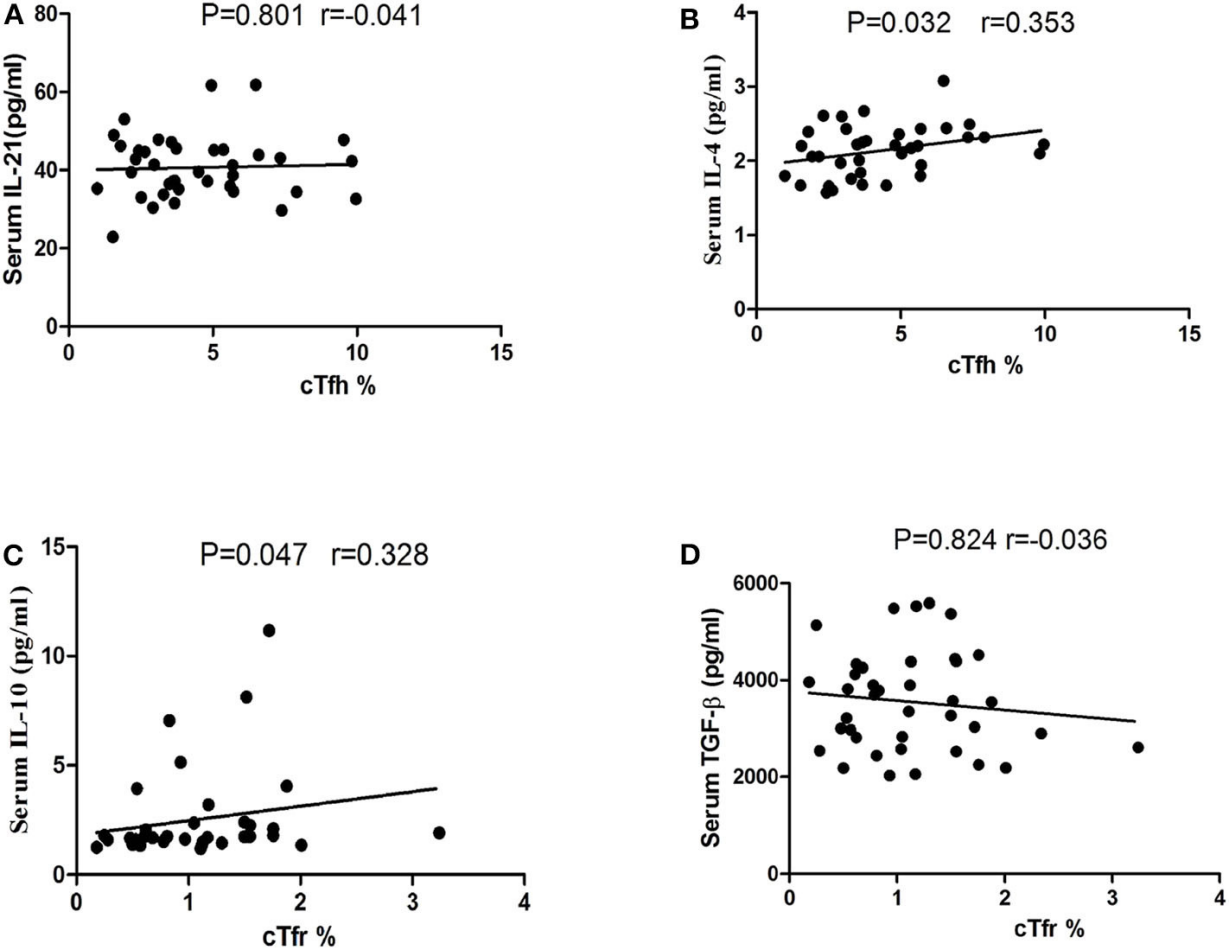
Correlation analysis of cTfh, cTfr with IL-21, IL-4, IL-10, and TGF-β in PD patients. **(A)** The correlation analysis of the percentages of cTfh cells with the serum IL-21 concentrations in PD patients (*P* = 0.801). **(B)** The correlation analysis of the percentages of cTfh cells with the serum IL-4 concentrations in PD patients (*P* = 0.032). **(C)** The correlation analysis of the percentages of cTfr cells with the serum IL-10 concentrations in PD patients (*P* = 0.047). **(D)** The correlation analysis of the percentages of cTfr cells with the serum TGF-β concentrations in PD patients (*P* = 0.824).

In our study, no significant correlations were found between the serum IL-21 concentrations and the number of cTfh in PD patients (*P* = 0.801 Figure 5A), however, we found that a positive trend of the correlation between the serum IL-4 concentrations and the number of cTfh in PD patients (*P* = 0.032, *r* = 0.353, Figure 5B). There was a positive trend of the correlation between the serum IL-10 concentrations and the number of cTfr in PD patients (*P* = 0.047, *r* = 0.328 Figure 5C), however, we didn't found the correlations between the serum TGF-β concentrations and the number of cTfr in PD patients (*P* = 0.824, Figure 5D).

### Correlations of the Levodopa Dosage With the cTfh Cells and B Cells in PD Patients

**Figure 6:**
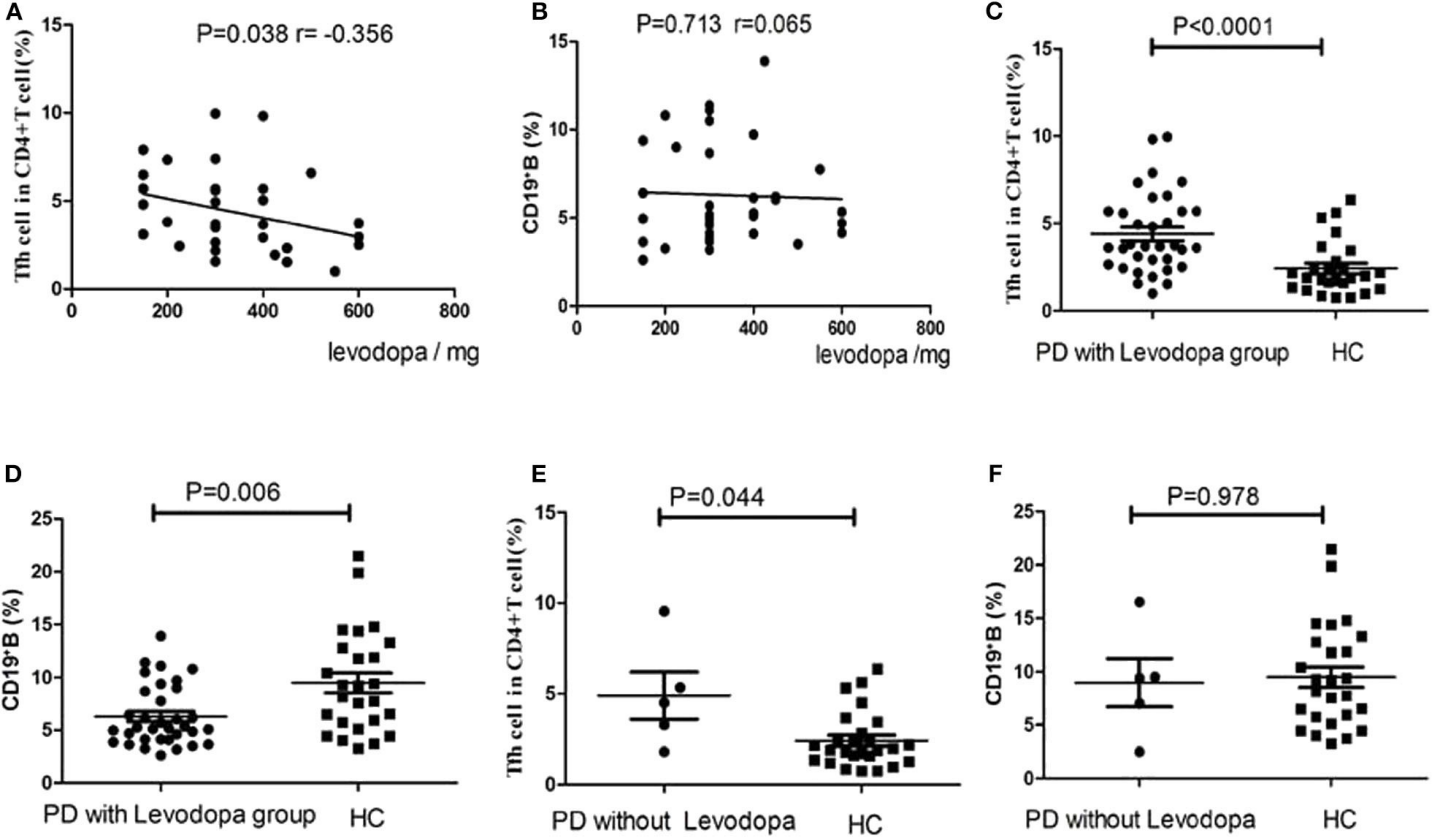
Correlation analysis of the levodopa dosage in PD patients with the cTfh cells and B cells. **(A)** The correlation analysis between levodopa dosage and the levels of Tfh cells (*P* = 0.038), **(B)** The correlation analysis between levodopa dosage and the levels of B cells (*P* = 0.713). **(C)** The comparison of the cTfh cells between PD with levodopa group and HCs (*P* < 0.0001). **(D)** The comparison of the B cells between PD with levodopa group and HCs (*P* = 0.006). **(E)** The comparison of the cTfh cells between PD without levodopa group and HCs (*P* = 0.044). **(F)** The comparison of the B cells between PD without levodopa group and HCs (*P* = 0.978).

We conducted a correlation analysis between the dosage of levodopa and the levels of cTfh cells and B cells in PD patients. There was a negative correlation trend between levodopa dosage and the levels of cTfh cells (*P* = 0.038, *r* = −0.356, Figure 6A), and no correlation was found with the levels of B cells (*P* = 0.713, *r* = 0.065, Figure 6B). In addition, we compared the cTfh cells and B cells in PD group with and without Levodopa to those in HCs, respectively. In PD with Levodopa group, the percentage of cTfh cells was significantly higher in PD patients (*P* < 0.0001, Figure 6C), the percentage of CD19+ B cells among PBMCs was significantly lower in PD patients (*P* = 0.006, Figure 6D). However, in PD without using Levodopa group, though the sample was small we also try to analyze, and the results showed that the cTfh cells was still higher than HCs (*P* = 0.044, Figure 6E), the percentage of CD19+ B cells among PBMCs was no obvious difference (*P* = 0.978, Figure 6F).

## Discussion

As a common neurological degenerative disease in the elderly, PD is a progressive disorder that affects movement. At present, the pathogenesis of PD remains incompletely understood. Mitochondrial dysfunction (19), cell apoptosis (20), ubiquitin-proteasome disorders (21), and oxidative stress (22) are thought to participate in PD pathogenesis. Additionally, immunity and inflammation have been proposed to be involved in PD (23). Chen et al. found that the proportion of CD4+ T cells among lymphocytes in patients with PD is decreased. Th1 cells and Th17 cells are increased significantly in the PD group, and the proportions of Th2 cells and regulatory T cells are decreased significantly (24). Studies have shown that the number of B lymphocytes in the peripheral blood of patients with PD is also decreased (25). Cytokines such as IL-2, IL-4, IL-10, IL-6, TNF-α, and IFN-γ are increased in the peripheral blood of patients with PD (26). In addition, Kim et al. showed that IL-10 concentrations are positively correlated with non-motor symptoms in PD (27). Up to now, studies on inflammation in PD have mainly focused on the detection of pro-inflammatory cytokines, such as TNF-α, IL-1β, IL-6, and IFN-γ, and anti-inflammatory cytokines, such as IL-10 and TGF-β, in the cerebrospinal fluid and peripheral blood of PD patients. Research on immunity in PD has mostly focused on the activation of microglia and astrocytes, and the imbalance of B lymphocytes and T lymphocytes and their subpopulations (28). Little research has been carried out to elucidate the role of humoral immunity in PD. The present study, to our knowledge, is the first to investigate the humoral immunity in PD by measuring the levels of cTfh and cTfr cells, and the related cytokines in PD patients. We found that the percentage of cTfh cells was increased and the percentage of cTfr cells among CD4^+^ T cells was decreased in PD patients compared to HCs, further analysis showed that the ratio of cTfh/cTfr was elevated, which indicating an activated humoral response state in PD. Furthermore, the concentration of serum IL-21, which is an effector molecular of cTfh cells, was a positive trend of the correlation with the H-Y stage and UPDRS-III score in PD patients, suggesting that IL-21 may be used as a marker to assess the severity of the disease.

Tfh cells can promote the generation of germinal centers, promote differentiation of B cells into plasma cells, and produce antibodies, which play an important role in humoral immune responses. Peripheral circulating Tfh cells express the same surface molecules as Tfh cells in lymphoid tissue and have the same function (29–31). In this study, cTfh cells were detected based on the CD4^+^CXCR5^+^PD-1^+^ expression pattern. Among these markers, PD-1 is a key phenotypic marker of Tfh cells. Due to the wide distribution of PD-1 ligands in the body, PD-1 plays an important role in regulating the immune response (32, 33). In addition, PD-1 on the surface of Tfh cells can also regulate the survival and affinity maturation of memory B cells and plasma cells at the germinal center and promote IL-21 secretion, leading to the production of more antibodies (34). Circulating Tfh cells were shown to be increased in PD patients compared to HCs, suggesting an imbalance of the humoral immune response in PD. It is speculated that this may be due to the continuous α-syn or other pathogenic factors stimulation in PD patients, which induces long-term activation of humoral immunity. Although no significant correlations were found between the percentage of cTfh cells and age, disease course, or disease severity (UPDRS-III) in the PD patients, studies with a large sample are needed for further verification of these results.

Tfr cells exert opposing functions to Tfh cells, inhibiting the formation of germinal centers and the production of antibodies. Although the mechanism is not clear, CTLA-4 and cytokines (IL-21, IL-4, IL-10, and TGF-β) are thought to be involved in the regulatory function of Tfr cells (35–38). The CD4^+^CXCR5^+^CD25^hi^CD127^low^ expression pattern was used to identify cTfr cells. Compared to HCs, the percentage of cTfr cells in PD patients in this study was not significantly altered, but showed a slightly decreasing trend, which suggests a possible decrease in cTfr cells in PD. A reduction of cTfr cells may lead to continuous activation of the humoral immune response, which may be related to the chronic progressive course of PD. The significantly increased cTfh/cTfr ratio in PD patients also confirms the presence of a persistent abnormal immune response in the progressive course of PD.

Consistent with the results of previous studies (25), the proportion of CD19^+^ B cells in PD patients was significantly reduced compared with that in HCs. T follicular helper cells can promote the differentiation of B cells into memory B cells and plasma cells in the germinal center, and IL-21 can assist this function. Theoretically, B cells will be increased as a response to the above changes in our study. However, beyond our expectation is that the number of CD19+B cells decreased which may be due to the following reasons. Firstly, what we detected in our experiment was the CD19+B lymphocytes in the peripheral blood, other subtype markers of B cells were not detected, which may not fully reflect the overall B cell level. In addition, the accumulation of B cells in the lymph tissues may also lead to this difference. Furthermore, the increase in Tfh cells and the decrease in B cells were inconsistent with normal assumptions in PD, suggesting that there may be other pathophysiological mechanisms participating in the interaction between Tfh cells and B cells in PD, which need further verification.

IL-4 and IL-21 are characteristic cytokines of Tfh cells (39), and research shows that IL-4 may be involved in the degeneration of dopamine neurons in PD (40). IL-10 and TGF-β which produced by Tfr cells can inhibit the response of Tfh cells and B cells in the germinal center (41). In this study, the serum concentrations of IL-4, IL-21, IL-10 and TGF-β were not significantly different between the PD patients and HCs. However, the serum concentration of IL-21 demonstrated a positive trend of the correlation with the severity of the disease in patients with PD. Previous studies have also shown that IL-21 is associated with the severity of diseases such as systemic lupus erythematosus, rheumatoid arthritis, type 1 diabetes, and Sjogren's syndrome (42). Thus, IL-21 may represent a useful biological indicator for monitoring the severity of PD as well as a new target for treatment. In addition, our study also found that the number of cTfh cells was a positive correlation trend with the serum concentration of IL-4 in PD patients, and the number of cTfr cells was a positive correlation trend with the serum concentration of IL-10 in PD patients. The cTfh cells and cTfr cells are positively correlated with their effect factors, which demonstrated their enhanced immunologic activities.

Levodopa has been shown to augment the T-folicular helper cell - B cell interaction in germinal centers (43). To further clarify whether the application of levodopa affects this interaction in our study, we analyzed the changes of cTfh cells and B cells under the administration of levodopa in PD patients. The results showed that there was a negative correlation trend between levodopa dosage and the levels of Tfh cells, and no correlation was found with the levels of B cells. In PD patients with and without Levodopa group, the percentage of cTfh cells was all higher than HCs. Based on the above analysis, there is no sufficient evidence demonstrating that the increase in percentage of cTfh and the decrease in percentage of B cells were surely caused by oral Levodopa. The negative correlation trend between levodopa dosage and the levels of Tfh cells also remind us that whether Levodopa plays a therapeutic role by modulating immune inflammatory response in addition to sole replacement therapy. Further research is needed.

## Conclusions

In summary, the percentage of cTfh cells was significantly increased in PD patients, and the percentage of cTfr cells showed a significant decreasing trend, the cTfh/cTfr ratio was further increased, which suggested a continuously activated humoral response state and may be involved in the chronic progression mechanism of PD. The serum IL-21 concentration was a positive trend of the correlation with the UPDRS-III score and H-Y stage in PD patients, which suggests that IL-21 may be a biomarker for monitoring the severity of PD and a possible target for disease intervention.

## Data Availability Statement

All datasets presented in this study are included in the article/Supplementary Material.

## Ethics Statement

The studies involving human participants were reviewed and approved by Ethics committee of the First Hospital of Jilin University. The patients/participants provided their written informed consent to participate in this study.

## Author Contributions

XZ, TJ, and YZ designed experiments and analyzed data. YZ revised the manuscript. All the authors collected data and contributed to the article and approved the submitted version.

## Conflict of Interest

The authors declare that the research was conducted in the absence of any commercial or financial relationships that could be construed as a potential conflict of interest.

## Supplementary Material

Supplementary Figure 1 The comparison of the UPDRS-III score between early PD group and middle-advanced PD group.

Supplementary Figure 2 The correlation of the cTfh/cTfr ratio and NMSS in PD patients after adjustment.

## References

[1] Kalia LV Lang AE. Parkinson's disease. Lancet. (2015) 386:896–912. 10.1016/S0140-6736(14)61393-3 25904081

[2] Braak H Del Tredici K Rub U de Vos RA Jansen Steur EN Braak E. Staging of brain pathology related to sporadic Parkinson's disease. Neurobiol Aging. (2003) 24:197–211. 10.1016/S0197-4580(02)00065-9 12498954

[3] Steiner JA Quansah E Brundin P. The concept of alpha-synuclein as a prion-like protein: ten years after. Cell Tissue Res. (2018) 373:161–73. 10.1007/s00441-018-2814-1 29480459 PMC6541204

[4] McGeer PL Itagaki S Boyes BE McGeer EG. Reactive microglia are positive for HLA-DR in the substantia nigra of Parkinson's and Alzheimer's disease brains. Neurology. (1988) 38:1285–91. 10.1212/WNL.38.8.1285 3399080

[5] Chen S Le WD Xie WJ Alexianu ME Engelhardt JI Siklos L. Experimental destruction of substantia nigra initiated by Parkinson disease immunoglobulins. Arch Neurol. (1998) 55:1075–80. 10.1001/archneur.55.8.1075 9708957

[6] Schaerli P Willimann K Lang AB Lipp M Loetscher P Moser B. CXC chemokine receptor 5 expression defines follicular homing T cells with B cell helper function. J Exp Med. (2000) 192:1553–62. 10.1084/jem.192.11.1553 11104798 PMC2193097

[7] Breitfeld D Ohl L Kremmer E Ellwart J Sallusto F Lipp M. Follicular B helper T cells express CXC chemokine receptor 5, localize to B cell follicles, and support immunoglobulin production. J Exp Med. (2000) 192:1545–52. 10.1084/jem.192.11.1545 11104797 PMC2193094

[8] Kim CH Rott LS Clark-Lewis I Campbell DJ Wu L Butcher EC. Subspecialization of CXCR5+ T cells: B helper activity is focused in a germinal center-localized subset of CXCR5+ T cells. J Exp Med. (2001) 193:1373–81. 10.1084/jem.193.12.1373 11413192 PMC2193300

[9] Wollenberg I Agua-Doce A Hernandez A Almeida C Oliveira VG Faro J. Regulation of the germinal center reaction by Foxp3+ follicular regulatory T cells. J Immunol. (2011) 187:4553–60. 10.4049/jimmunol.1101328 21984700

[10] Sage PT Sharpe AH. T follicular regulatory cells in the regulation of B cell responses. Trends Immunol. (2015) 36:410–8. 10.1016/j.it.2015.05.005 26091728 PMC4508020

[11] Wang X Yang C Xu F Qi L Wang J Yang P. Imbalance of circulating Tfr/Tfh ratio in patients with rheumatoid arthritis. Clin Exp Med. (2019) 19:55–64. 10.1007/s10238-018-0530-5 30284646

[12] Wen Y Yang B Lu J Zhang J Yang H Li J. Imbalance of circulating CD4(+). CXCR5(+). FOXP3(+). Tfr-like cells and CD4(+). CXCR5(+). FOXP3(-). Tfh-like cells in myasthenia gravis. Neurosci Lett. (2016) 630:176–82. 10.1016/j.neulet.2016.07.049 27473945

[13] Wang X Zhu Y Zhang M Hou J Wang H Jiang Y. The shifted balance between circulating follicular regulatory T cells and follicular helper T cells in patients with ulcerative colitis. Clin Sci. (2017) 131:2933–45. 10.1042/CS20171258 29109300

[14] Yan L Li Y Li Y Wu X Wang X Wang L Increased circulating Tfh to Tfr ratio in chronic renal allograft dysfunction: a pilot study. BMC Immunol. (2019) 20:26 10.1186/s12865-019-0308-x 31382877 PMC6683539

[15] Postuma RB Berg D Stern M Poewe W Olanow CW Oertel W. MDS clinical diagnostic criteria for Parkinson's disease. Mov Disord. (2015) 30:1591–601. 10.1002/mds.26424 26474316

[16] The Unified Parkinson's disease rating scale (UPDRS): status and recommendations. Mov Disord. (2003) 18:738–50. 10.1002/mds.10473 12815652

[17] Goetz CG Poewe W Rascol O Sampaio C Stebbins GT Counsell C. Movement Disorder Society Task Force report on the Hoehn and Yahr staging scale: status and recommendations. Mov Disord. (2004) 19:1020–8. 10.1002/mds.20213 15372591

[18] Chaudhuri KR Martinez-Martin P Brown RG Sethi K Stocchi F Odin P. The metric properties of a novel non-motor symptoms scale for Parkinson's disease: Results from an international pilot study. Mov Disord. (2007) 22:1901–11. 10.1002/mds.21596 17674410

[19] Bose A Beal MF. Mitochondrial dysfunction in Parkinson's disease. J Neurochem. (2016) 139 (Suppl. 1):216–31. 10.1111/jnc.13731 27546335

[20] Tatton WG Chalmers-Redman R Brown D Tatton N. Apoptosis in Parkinson's disease: signals for neuronal degradation. Ann Neurol. (2003) 53 (Suppl. 3):S61–70; discussion S70-62. 10.1002/ana.10489 12666099

[21] Betarbet R Sherer TB Greenamyre JT. Ubiquitin-proteasome system and Parkinson's diseases. Exp Neurol. (2005) 191 (Suppl. 1):S17–27. 10.1016/j.expneurol.2004.08.021 15629758

[22] Puspita L Chung SY Shim JW. Oxidative stress and cellular pathologies in Parkinson's disease. Mol Brain. (2017) 10:53. 10.1186/s13041-017-0340-9 29183391 PMC5706368

[23] Mosley RL Hutter-Saunders JA Stone DK Gendelman HE. Inflammation and adaptive immunity in Parkinson's disease. Cold Spring Harb Perspect Med. (2012) 2:a009381. 10.1101/cshperspect.a009381 22315722 PMC3253034

[24] Chen Y Qi B Xu W Ma B Li L Chen Q. Clinical correlation of peripheral CD4+cell subsets, their imbalance and Parkinson's disease. Mol Med Rep. (2015) 12:6105–11. 10.3892/mmr.2015.4136 26239429

[25] Stevens CH Rowe D Morel-Kopp MC Orr C Russell T Ranola M. Reduced T helper and B lymphocytes in Parkinson's disease. J Neuroimmunol. (2012) 252:95–9. 10.1016/j.jneuroim.2012.07.015 22910543

[26] Brodacki B Staszewski J Toczylowska B Kozlowska E Drela N Chalimoniuk M. Serum interleukin (IL-2, IL-10, IL-6, IL-4), TNFalpha, and INFgamma concentrations are elevated in patients with atypical and idiopathic parkinsonism. Neurosci Lett. (2008) 441:158–62. 10.1016/j.neulet.2008.06.040 18582534

[27] Kim R Kim HJ Kim A Jang M Kim A Kim Y. Peripheral blood inflammatory markers in early Parkinson's disease. J Clin Neurosci. (2018) 58:30–3. 10.1016/j.jocn.2018.10.079 30454693

[28] Joshi N Singh S. Updates on immunity and inflammation in Parkinson disease pathology. J Neurosci Res. (2018) 96:379–90. 10.1002/jnr.24185 29072332

[29] Schmitt N Bentebibel SE Ueno H. Phenotype and functions of memory Tfh cells in human blood. Trends Immunol. (2014) 35:436–42. 10.1016/j.it.2014.06.002 24998903 PMC4152409

[30] Chevalier N Jarrossay D Ho E Avery DT Ma CS Yu D. CXCR5 expressing human central memory CD4 T cells and their relevance for humoral immune responses. J Immunol. (2011) 186:5556–68. 10.4049/jimmunol.1002828 21471443

[31] Morita R Schmitt N Bentebibel SE Ranganathan R Bourdery L Zurawski G. Human blood CXCR5(+). CD4(+). T cells are counterparts of T follicular cells and contain specific subsets that differentially support antibody secretion. Immunity. (2011) 34:108–21. 10.1016/j.immuni.2010.12.012 21215658 PMC3046815

[32] Greisen SR Rasmussen TK Stengaard-Pedersen K Hetland ML Horslev-Petersen K Hvid M. Increased soluble programmed death-1 (sPD-1) is associated with disease activity and radiographic progression in early rheumatoid arthritis. Scand J Rheumatol. (2014) 43:101–8. 10.3109/03009742.2013.823517 24182347

[33] Weissferdt A Fujimoto J Kalhor N Rodriguez J Bassett R Wistuba II. Expression of PD-1 and PD-L1 in thymic epithelial neoplasms. Mod Pathol. (2017) 30:826–33. 10.1038/modpathol.2017.6 28281549

[34] Good-Jacobson KL Szumilas CG Chen L Sharpe AH Tomayko MM Shlomchik MJ. PD-1 regulates germinal center B cell survival and the formation and affinity of long-lived plasma cells. Nat Immunol. (2010) 11:535–42. 10.1038/ni.1877 20453843 PMC2874069

[35] Wing JB Ise W Kurosaki T Sakaguchi S. Regulatory T cells control antigen-specific expansion of Tfh cell number and humoral immune responses via the coreceptor CTLA-4. Immunity. (2014) 41:1013–25. 10.1016/j.immuni.2014.12.006 25526312

[36] Sage PT Paterson AM Lovitch SB Sharpe AH. The coinhibitory receptor CTLA-4 controls B cell responses by modulating T follicular helper, T follicular regulatory, and T regulatory cells. Immunity. (2014) 41:1026–39. 10.1016/j.immuni.2014.12.005 25526313 PMC4309019

[37] Ding L Linsley PS Huang LY Germain RN Shevach EM. IL-10 inhibits macrophage costimulatory activity by selectively inhibiting the up-regulation of B7 expression. J Immunol. (1993) 151:1224–34. 7687627

[38] McCarron MJ Marie JC. TGF-beta prevents T follicular helper cell accumulation and B cell autoreactivity. J Clin Investig. (2014) 124:4375–86. 10.1172/JCI76179 25157822 PMC4191003

[39] McGuire HM Vogelzang A Warren J Loetsch C Natividad KD Chan TD. IL-21 and IL-4 collaborate to shape T-dependent antibody responses. J Immunol. (2015) 195:5123–35. 10.4049/jimmunol.1501463 26491200

[40] Bok E Cho EJ Chung ES Shin WH Jin BK. Interleukin-4 contributes to degeneration of dopamine neurons in the lipopolysaccharide-treated Substantia Nigra *in vivo*. Exp Neurobiol. (2018) 27:309–19. 10.5607/en.2018.27.4.309 30181693 PMC6120964

[41] Stebegg M Kumar SD Silva-Cayetano A Fonseca VR Linterman MA Graca L. Regulation of the germinal center response. Front Immunol. (2018) 9:2469. 10.3389/fimmu.2018.02469 30410492 PMC6209676

[42] Long D Chen Y Wu H Zhao M Lu Q. Clinical significance and immunobiology of IL-21 in autoimmunity. J Autoimmunity. (2019) 99:1–14. 10.1016/j.jaut.2019.01.013 30773373

[43] Papa I Saliba D Ponzoni M Bustamante S Canete PF Gonzalez-Figueroa P. TFH-derived dopamine accelerates productive synapses in germinal centres. Nature. (2017) 547:318–23. 10.1038/nature23013 28700579 PMC5540173

[44] Zhao X Jin T Zheng C Ma D Zhang Y Imbalance of circulating Tfh/Tfr cells in patients with Parkinson's disease. Preprint. (2020). 10.21203/rs.3.rs-28078/v1 PMC7573556 33123078

