# Supplementary figures and images for: Imbalance of Circulating Tfh/Tfr Cells in Patients With Parkinson's Disease
Source: Front Neurol. 2020 Oct 2;11:572205. doi: 10.3389/fneur.2020.572205 (PMC7573556; doi:10.3389/fneur.2020.572205)

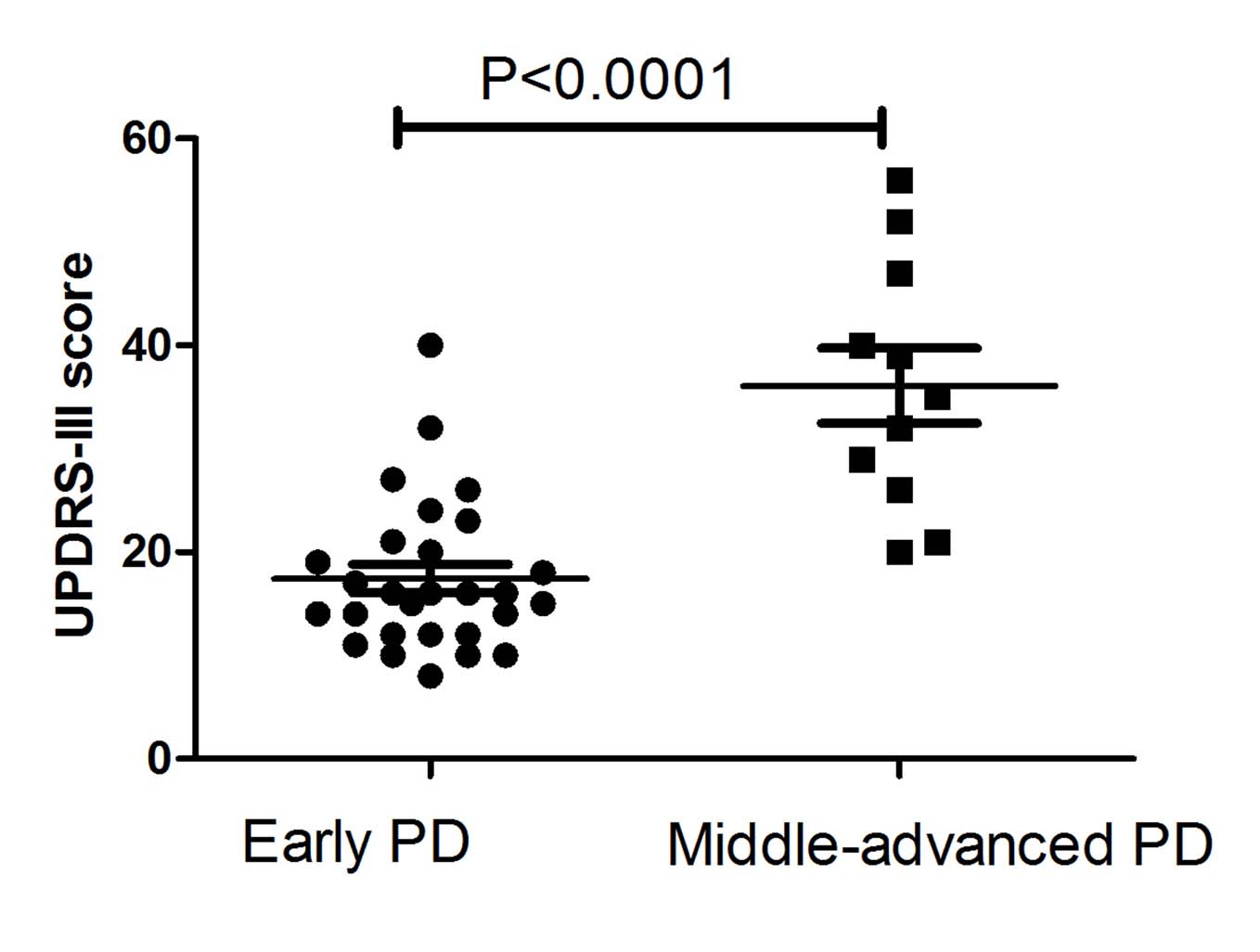

Supplement: Supplementary Figure 1 — The comparison of the UPDRS-III score between early PD group and middle-advanced PD group. [file Image_1.JPEG]

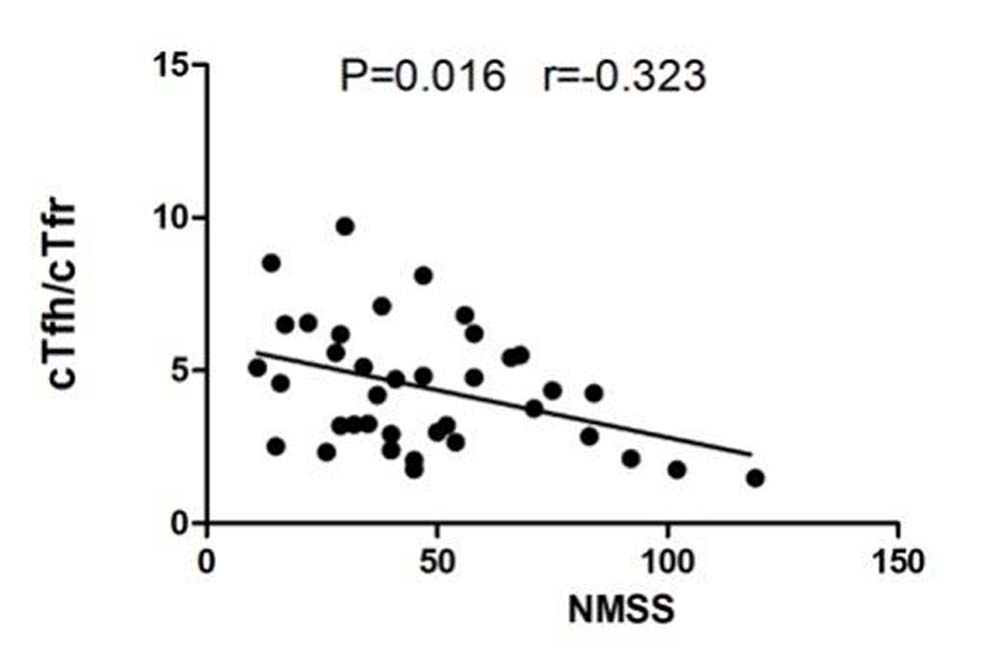

Supplement: Supplementary Figure 2 — The correlation of the cTfh/cTfr ratio and NMSS in PD patients after adjustment. [file Image_2.jpg]
